# Daily Max Simplified Wet-Bulb Globe Temperature and its Climate Networks for Teleconnection Study, 1940–2022

**DOI:** 10.1038/s41597-025-04933-w

**Published:** 2025-04-07

**Authors:** Yan Liu, Changqing Song, Sijing Ye, Jiaying Lv, Peichao Gao

**Affiliations:** State Key Laboratory of Earth Surface Processes and Hazards Risk Governance, Faculty of Geographical Science, Beijing Normal University, Beijing, 100875 China

**Keywords:** Natural hazards, Atmospheric dynamics

## Abstract

As global warming intensifies, extreme heat events, especially those occurring simultaneously or sequentially in multiple regions, are becoming more frequent. This highlights the growing need to analyze heat stress from the perspectives of human health and spatiotemporal correlations. Wet-Bulb Globe Temperature (WBGT) is a well-established heat stress indicator closely linked to human health. However, its reliance on specialized measurements and resource-intensive computations limits its widespread use, particularly for researchers without an earth sciences background. To address this, we adopted a simplified WBGT (sWBGT), which effectively simulates human cooling through sweating, to generate a global 2° resolution dataset of daily maximum sWBGT from 1940 to 2022. This dataset fills a critical gap in long-term, global-scale heat stress data. Additionally, we employed climate network methods to innovatively explore teleconnections of extreme heat events, providing a tool to reveal their spatiotemporal relationships and supporting the development of effective health protection strategies.

## Background & Summary

As the trend of global warming intensifies, the adverse effects of heat extremes on human health have become increasingly significant and cannot be ignored^1–4^. Future projections from climate model simulation predictions indicate that, in the future, humans will have to confront more severe heat stress challenges^5–9^. Therefore, it is crucial to select an appropriate indicator to measure the impact of heat stress on human health. Compared to considering only temperature, incorporating both temperature and humidity into the assessment can reveal the actual impact of heat stress on human health more accurately^10–12^. Numerous heat indices have been developed by researchers to evaluate the combined effects of temperature and humidity on human health^13,14^. For example, the Heat Index (HI) has found wide applicability in estimating human heat stress. It has proven useful both in research on future climate change impacts and operationally for issuing heat advisories by the U.S. National Weather Service (NWS)^15^. The Standard Effective Temperature (SET) is also widely used in thermal comfort and environmental research. It reflects the heat exchange mechanisms between the human body and its surroundings, and it has been incorporated into U.S. national standards^16^. The Universal Thermal Climate Index (UTCI), developed by the International Society of Biometeorology (ISB), evaluates the human body’s thermal response under varying climatic conditions, considering a broader range of environmental factors that contribute to thermal stress^17^. Among these, the Wet-Bulb Globe Temperature (WBGT), a weighted average of ambient, wet-bulb, and globe temperatures, is widely recognized and frequently employed in heat stress assessments^18,19^. This prominence stems from its comprehensive integration of multiple meteorological variables, such as temperature, humidity, wind speed, and solar radiation, into a single index^20^. Compared to simpler indices, WBGT captures the interactive effects of these factors on human heat stress, making it a reliable measure for occupational health, military operations, and athletic performance^21^. Its strong theoretical foundation and empirical validation further solidify its status as the standard for heat stress assessment in national and international guidelines^18,22^.

Despite its importance, WBGT faces several challenges in practical applications. These include: (1) Practical challenges in WBGT measurement: Direct measurement requires costly instruments and skilled operators^23^, making it impractical for routine meteorological observations^24^. Consequently, obtaining large-scale, multi-temporal, and high-accuracy WBGT data has been a longstanding challenge. To address this, researchers have developed physical models based on the energy balance of WBGT sensors, using standard meteorological station data to calculate WBGT^25–27^. Among these, Liljegren’s model is well-validated and considered the “gold standard” for WBGT calculation^28–30^. However, this introduces additional challenges. (2) Resource-intensive exact calculations: While precise WBGT calculations (e.g., via the Liljegren method) yield accurate results, they involve iterative solutions to nonlinear equations, such as those for natural wet-bulb and globe temperatures^26^, demanding significant computational resources and expertise^30^. (3) Limited availability of meteorological data: WBGT computation relies on variables like air temperature, relative humidity, wind speed, and solar radiation. In less-developed regions, these data are often sparse; even in climate models, variables such as wind speed and radiation lack sufficient spatiotemporal resolution or exhibit high uncertainty^31,32^. (4) Contextual limitations of WBGT: Designed primarily for unshaded outdoor heat stress, WBGT is less suited to indoor or shaded environments—common in daily life—where wind speed and solar radiation effects are diminished^33,34^.

To address these challenges, scholars have developed a simplified WBGT (sWBGT) method by simplifying the WBGT formula and its variables, aiming to make WBGT calculation more accessible while ensuring accurate heat stress assessment^35–38^. Compared to WBGT, sWBGT offers two key advantages: (1) Convenience and effectiveness of sWBGT: By simplifying assumptions (e.g., fixed radiation or low wind speed) and reducing variable requirements, sWBGT enables rapid and practical heat stress estimation^23,30^. Though it sacrifices some precision, its effectiveness has been validated across various applications^39^. (2) Practical value of sWBGT: sWBGT lowers the barrier to use, empowering workers, employers, and local authorities to manage heat stress more effectively^31,40^, particularly in resource-constrained regions, aligning with our goal of producing a global gridded dataset.

One common simplification replaces the globe temperature with the dry-bulb temperature to describe heat stress in indoor or well-shaded conditions^23,33,34,41^. Building on this, Li *et al*. further substituted the natural wet-bulb temperature with the isobaric wet-bulb temperature, better simulating heat reduction through sweating^32^. This led to the development of sWBGT, a convenient and efficient index for assessing heat exposure, widely used to evaluate the impact of heat stress on labor^42–44^. Although sWBGT holds significant value in fields like climate change, public health, and socioeconomics, no global-scale gridded dataset based on this index currently exists. Thus, we aim to provide a sWBGT-based dataset to facilitate its use by scholars, saving time and computational resources. This dataset fills a gap in existing research, providing a new tool for global heat stress analysis.

With the intensification of global warming, the frequency of extreme heat events has significantly increased^45^. These events are not only occurring in single regions but often concurrently or sequentially in multiple regions^46^. Compared to individual events, the concurrent or sequential occurrence of extreme heat events in different regions has a more profound impact, particularly on agriculture, power systems, and infrastructure capacity^46^. For this reason, the spatiotemporal correlations between extreme heat events have garnered significant attention from researchers^47,48^. Studying the spatiotemporal correlations of these events and analyzing the underlying propagation mechanisms is of great importance in coping with heat stress^1,49,50^. Furthermore, teleconnection, as a phenomenon that describes the persistent connection of climate anomalies between distant regions, is typically caused by large-scale atmospheric or oceanic activities that involve long-range transport of energy and materials^51,52^. Traditional methods for analyzing teleconnections, such as Principal Component Analysis, can capture the main patterns but cannot identify the propagation direction or key nodes. The climate network approach offers unique advantages in this regard. In climate networks, geographical locations are represented as nodes, and the connections between nodes are generated using statistical measures such as cross-correlation or mutual information between the time series of meteorological variables (e.g., temperature, precipitation)^53–56^. These networks reveal pairs of strongly correlated nodes, the propagation direction of variables, and the time-lagged relationships between events. This method has been applied to explore climate teleconnection mechanisms and predict extreme events^50,56–58^. However, most climate network-based research on heat stress teleconnections has thus far focused on local or regional scales^50,58^, with few studies at the global scale. Yet the climate system, as a complex system, can exhibit different degrees of complexity and distinct evolutionary patterns across spatial scales^59^, underscoring the need for broader-scale investigations. On the other hand, the climate network is a method that requires significant computational resources and time, which results in a relatively high threshold for implementation. Therefore, this study builds a climate network on a global scale based on sWBGT data, aiming to provide data support for the analysis of heat stress teleconnections. This provision is intended to serve as a reference for researchers and to conserve time and economic resources required for calculating climate networks. At the same time, we conducted a simple analysis of climate network data, providing some basic reference indicators, such as in-degree, out-degree, and degree centrality.

## Methods

### Overview

**Fig. 1:**
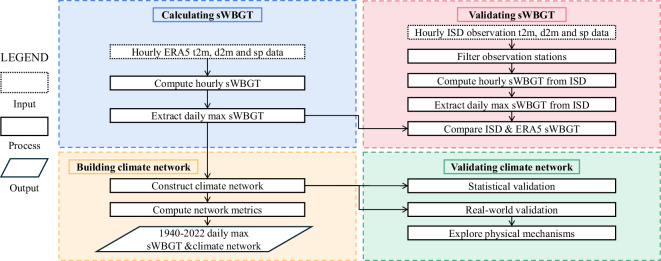
The framework for developing the sWBGT and climate network dataset.

We utilized a reanalysis dataset to compute global hourly values of simplified wet-bulb globe temperature (sWBGT) from January 1940 to December 2022. From this, we derived daily maximum sWBGT values at a spatial resolution of 2° × 2°. Based on these daily maxima, we constructed climate networks for selected years (1940, 1945,…, 2020) and calculated related network metrics. Figure 1 depicts the workflow of this study. Additionally, we provide Python scripts to facilitate the replication of these computations on other datasets.

### Reanalysis data

We used the ‘ERA5 hourly data on single levels from 1940 to present’ dataset to calculate the sWBGT^60^. From this dataset, we utilized three hourly variables: (1) two-meter air temperature, (2) two-meter dew point temperature, and (3) surface pressure. Although the spatial resolution of atmospheric reanalysis variables in this dataset is 0.25° × 0.25°, to facilitate global-scale computations, we chose to download these three variables at a coarser spatial resolution of 2° × 2°.

### Calculating simplified wet-bulb globe temperatures

The WBGT can be calculated using the natural wet-bulb temperature (${T}_{w}$), black globe temperature (${T}_{g}$), and the dry-bulb temperature (ambient temperature, ${T}_{a}$) as follows^18^:

1 $${WBGT}=0.7\cdot {T}_{w}+0.2\cdot {T}_{g}+0.1\cdot {T}_{a}$$

As previously mentioned, since ${T}_{w}$ and ${T}_{g}$ are not standard meteorological variables and their observational data are difficult to obtain directly, they usually need to be estimated through modeling. Among the various modeling approaches, the model proposed by Liljegren, which is based on an energy balance, is considered to be relatively accurate. The equations are as follows^26^:

2 $${T}_{w}={T}_{a}-\frac{\Delta H}{{C}_{p}}\frac{{M}_{H2O}}{{M}_{A{ir}}}{\left(\frac{{P}_{r}}{{S}_{c}}\right)}^{0.56}\left(\frac{{e}_{w}-{e}_{a}}{P-{e}_{w}}\right)+\frac{\Delta {F}_{{net}}}{{Ah}}$$

Where $\Delta H$ represents the heat of vaporization; ${C}_{p}$ is the specific heat of dry air at constant pressure; ${M}_{H2O}$ and ${M}_{A{ir}}$ denote the molecular weight of water vapor and dry air, respectively; ${P}_{r}$ is the Prandtl number, and ${S}_{c}$ is the Schmidt number. ${e}_{w}$ and ${e}_{a}$ represent the water vapor pressure at the wet-bulb surface and in the ambient air, respectively; $P$ is the barometric pressure; $A$ is the surface area of the wick; $h$ is the convective heat transfer coefficient. The term $\Delta {F}_{{net}}$ represents the net radiant heat flux from the environment to the wick and is a function of ${T}_{w}^{4}$, which means it cannot be directly solved analytically^26^.

3 $${T}_{g}^{4}=\frac{1}{2}\left(1+{\varepsilon }_{a}\right){T}_{a}^{4}-\frac{{\rm{h}}}{{\varepsilon }_{g}\sigma }\left({T}_{g}-{T}_{a}\right)+\frac{S}{2{\varepsilon }_{g}\sigma }\left(1-{a}_{g}\right)\left[1+\left(\frac{1}{2\cos \left(\theta \right)}-1\right){f}_{{dir}}+{a}_{{sfc}}\right]$$

where ${\varepsilon }_{g}$ and ${\varepsilon }_{a}$ are the globe and atmospheric emissivity, respectively; $S$ represents the total horizontal solar irradiance; $\sigma$ is the Stefan-Boltzmann constant; ${f}_{{dir}}$ is the fraction of $S$ due to the direct beam radiation; and ${a}_{{sfc}}$ is the surface albedo. Similarly, it can be observed that due to the presence of the ${T}_{g}^{4}$ term, an analytical solution is not feasible.

Since ${T}_{g}$ has a relatively small weight in the WBGT calculation and its computation is complex, some researchers have chosen to replace ${T}_{g}$ with ${T}_{a}$. Given that ${T}_{g}$ is influenced by solar radiation and wind speed, this simplification is only applicable under indoor or well-shaded thermal conditions. In such cases, sWBGT can be calculated as follows^33^:

4 $${sWBGT}=0.7\cdot {T}_{w}+0.3\cdot {T}_{a}$$

In the calculation of sWBGT, ${T}_{w}$ is assigned a substantial weight of 0.7, making it a crucial component. ${T}_{w}$ is traditionally measured using a wet-bulb psychrometer. Due to the challenge of directly obtaining the natural wet-bulb temperature from climate model outputs, previous studies have explored several approximate methods for its calculation^61^. In this context, we utilize the “isobaric wet-bulb temperature” as a proxy for ${T}_{w}$, defined as the temperature an air parcel would attain after becoming saturated with water vapor that has evaporated into it, with the entire air-water system held at constant pressure and insulated from the environment. This measure is more directly related to the efficiency of human cooling through perspiration and is, therefore, employed in our calculation of sWBGT. Specifically, ${T}_{w}$ can be calculated by solving the function as fellow^32^:

5 $${c}_{pa}{T}_{w}+{L}_{v}{r}_{s}{T}_{w}={c}_{{pa}}T+{L}_{v}$$

Where ${c}_{pa}$ is the specific heat capacity of air under constant pressure. ${L}_{v}$ is the latent heat of evaporation of water. ${r}_{s}$ is the equilibrium specific humidity, dependent on the saturation water vapor pressure at temperature ${T}_{w}$. The relevant Python scripts provided by Li *et al*. utilize air temperature, dew point temperature, and surface temperature. The sWBGT is calculated hourly, and the daily maximum is selected for each day.

### Constructing climate networks

In this study, we constructed climate networks using the approach adapted from Liu *et al*. for selected years from 1940 to 2020 at five-year intervals (i.e., 1940, 1945, 1950,…, 2015, 2020)^53^. Specifically, we first removed the values for February 29th from all leap years in the SWBGT dataset and eliminated linear and seasonal trends from the global data. Each grid point was treated as a node, resulting in a total of 16380 nodes globally. We then calculated the cross-correlation between each pair of nodes using the following formula:

6 $${C}_{i,j}^{y}\left(\sigma \right)=\frac{\left\langle {T}_{i}\left(d\right){T}_{j}\left(d+\sigma \right)\right\rangle -\left\langle {T}_{i}\left(d\right)\right\rangle \left\langle {T}_{j}\left(d+\sigma \right)\right\rangle }{\sqrt{\left\langle {\left({T}_{i}\left(d\right)-\left\langle {T}_{i}\left(d\right)\right\rangle \right)}^{2}\right\rangle }\times \sqrt{\left\langle {\left({T}_{j}\left(d+\sigma \right)-\left\langle {T}_{j}\left(d+\sigma \right)\right\rangle \right)}^{2}\right\rangle }}$$

where ${C}_{i,j}^{y}\left(\sigma \right)$ represents the cross-correlation between node $i$ and node $j$ at the starting year $y$ with time lag $\sigma$, and $\sigma \in \left[0,{\sigma }_{\max }\right]$ with ${\sigma }_{\max }=200{days}$. Here, ${T}_{i}\left(d\right)$ denotes the 365-day time series of node $i$, and ${T}_{j}\left(d+\sigma \right)$ is the time series of node $j$ shifted by $\sigma$ days. The link weights ${C}_{i,j}^{y}\left({\sigma }_{0}\right)$ represent the maximum absolute value of the cross-correlation between nodes $i$ and $j$ at a specific time lag ${\sigma }_{0}$, indicating the strength of the linear relationship between the two nodes’ time series at that lag. In the context of extreme heat stress (e.g., SWBGT), a large $\left|{C}_{i,j}^{y}\left({\sigma }_{0}\right)\right|$ suggests a strong linear relationship between the heat stress variations of the two regions. For each pair of nodes in each starting year $y$, 2 ${\sigma }_{\max }+1$ cross-correlations are computed, and the maximum absolute value is identified along with the corresponding time lag ${\sigma }_{0}$. The direction of the link is determined based on the sign of ${\sigma }_{0}$: if ${\sigma }_{0} > 0$, the direction of the link is from $i$ to $j$; conversely, when ${\sigma }_{0} < 0$, the direction of the link is from $j$ to $i$.

To evaluate the significance of these links, we calculate the strength of the link ${W}_{i,j}^{y}$, defines as:

7 $${W}_{i,j}^{y}=\frac{{C}_{i,j}^{y}\left({\sigma }_{0}\right)-\left\langle {C}_{i,j}^{y}\left(\sigma \right)\right\rangle }{{std}\left({C}_{i,j}^{y}\left(\sigma \right)\right)}$$

This link strength ${W}_{i,j}^{y}$ is a normalized measure that quantifies how significantly the peak correlation stands out from the overall cross-correlation function by subtracting the mean and dividing by the standard deviation of ${C}_{i,j}^{y}\left(\sigma \right)$ over all lags. A larger ${W}_{i,j}^{y}$ indicates a more prominent and statistically significant link between nodes $i$ and $j$, suggesting a robust teleconnection in heat stress variations, while a small or near-zero value implies the peak does not exceed the background correlation significantly, indicating a weaker teleconnection. Link density, which reflects the compactness of connections within the network, was set to 0.05% following previous studies^62,63^. To achieve this, we used the 95th percentile of the absolute values of ${C}_{i,j}^{y}\left({\sigma }_{0}\right)$ as the threshold for selecting connections. Values of ${C}_{i,j}^{y}\left({\sigma }_{0}\right)$ and ${W}_{i,j}^{y}$ below this threshold were set to zero. This moderate density allows us to capture the primary structures and patterns within the network while avoiding excessive noise and complexity.

### Calculating network measures

After constructing the climate networks, we calculated several network measures to analyze the structural properties and dynamics of the networks. These measures provide insights into how nodes (representing geographical regions) influence or are influenced by others within the heat stress teleconnection network. The key measures are defined as follows:

#### In-degree and Out-degree

The in-degree of a node represents the number of directed links incoming to that node from other nodes, indicating how many other nodes can influence its heat stress variations. Conversely, the out-degree represents the number of directed links outgoing from the node, showing how many other nodes can be affected by its heat stress variations. Larger values of in-degree or out-degree suggest that the node either receives or exerts influence over a greater number of nodes. Mathematically, these are calculated as:

8 $${I}_{i}^{y}={\sum }_{j}{A}_{j,i}^{y},{O}_{i}^{y}={\sum }_{j}{A}_{i,j}^{y}$$

where ${A}_{i,j}^{y}$=1 if there is a directed link from $i$ to $j$, and 0 otherwise.

#### In-weights and Out-weights

The in-weights sum the weights of all incoming links to a node, quantifying the total strength of influences received from other nodes in terms of their heat stress correlations. Similarly, the out-weights sum the weights of all outgoing links, indicating the total strength of influences exerted on other nodes. A larger value reflects greater overall consistency in heat stress changes between the node and others in the network. These are calculated as:

9 $${IN}\left({C}_{i}^{y}\right)={\sum }_{j}{A}_{j,i}^{y}{C}_{j,i}^{y}\left({\sigma }_{0}\right),{OUT}\left({C}_{i}^{y}\right)={\sum }_{j}{A}_{i,j}^{y}{C}_{i,j}^{y}\left({\sigma }_{0}\right)$$

#### In-strength and Out-strength

The in-strength sums the link strengths of all incoming links, reflecting the statistical significance of the heat stress signals received by the node from others. The out-strength sums the link strengths of all outgoing links, indicating the statistical significance of the heat stress signals sent to other nodes. Larger values imply that the node’s connections are more pronounced and likely represent robust teleconnections.These are calculated using the normalized link strengths ${W}_{i,j}^{y}$:

10 $${IN}\left({W}_{i}^{y}\right)={\sum }_{j}{A}_{j,i}^{y}{W}_{j,i}^{y},{OUT}\left({W}_{i}^{y}\right)={\sum }_{j}{A}_{i,j}^{y}{W}_{i,j}^{y}$$

#### Degree centrality

Degree centrality is the sum of a node’s in-degree and out-degree, indicating its overall connectivity within the network. Nodes with high degree centrality act as hubs, connecting multiple distant regions and playing a pivotal role in the dynamics of heat stress teleconnections. It is calculated as:

11 $${{DC}}_{i}^{y}={I}_{i}^{y}+{O}_{i}^{y}$$

#### Area weighted connectivity (AWC)

AWC measures the proportion of the Earth’s surface area covered by a node’s connections, accounting for the geographical extent of its teleconnection influence^64^. A larger AWC suggests that the node’s influence extends across broader regions, enhancing its overall impact on the network.The formula is:

12 $${{AWC}}_{i}^{y}=\frac{{\sum }_{j}{A}_{i,j}^{y}\cos {\lambda }_{j}+{\sum }_{j}{A}_{j,i}^{y}\cos {\lambda }_{j}}{{\sum }_{j}\cos {\lambda }_{j}}$$

${\lambda }_{j}$ represents the latitude of node $j$. Since the area corresponding to each node is proportional to the cosine of its latitude, the area-weighted connectivity represents the proportion of the node’s connection area relative to the Earth’s surface area. Consequently, the value of ${{AWC}}_{i}^{y}$ ranges between 0 and 1.

#### Network divergence (ND)

ND is calculated as the difference between a node’s out-degree and in-degree, indicating whether the node acts primarily as a source or a sink of heat stress signals. A positive ND suggests the node is a source, influencing other nodes, while a negative ND indicates it is a sink, receiving influences from others. It is calculated as:

13 $${{ND}}_{i}^{y}={O}_{i}^{y}-{I}_{i}^{y}$$

These network measures collectively provide a comprehensive understanding of the teleconnection patterns in heat stress across different regions, highlighting the roles and significance of various nodes within the global climate network.

### sWBGT data accuracy evaluation

To assess the accuracy of the sWBGT data, we selected the validation year based on the availability and completeness of meteorological station data. A station was considered eligible if it met the following conditions:

1. Provided all three key meteorological variables required for sWBGT computation: air temperature, dew point temperature, and surface air pressure.
2. Had at least 6,132 hours of observational data (covering 70% of the year).
3. Recorded data at precise hourly intervals, with observations within a 10-minute deviation from the hour adjusted to the nearest whole hour.

Based on these criteria, we acquired data from 13,474 global stations for the year 2022, sourced from the Integrated Surface Database maintained by the National Oceanic and Atmospheric Administration (NOAA)^65^. In total, 1,858 stations worldwide met our selection criteria, providing a well-distributed representation across different continents. This ensures that our sWBGT values are validated under diverse geographic and climatic conditions.

We employed four statistical metrics to assess the accuracy of the sWBGT data calculated from these observations: the coefficient of determination (R²), mean absolute error (MAE), bias, and root mean square error (RMSE). The metrics are calculated as follows:

Coefficient of Determination (R²): This metric provides a measure of how well the observed outcomes are replicated by our data, defined as:

14 $${R}^{2}=1-\frac{{\sum }_{i=1}^{n}{\left({y}_{i}-\hat{{y}_{i}}\right)}^{2}}{{\sum }_{i=1}^{n}{\left({y}_{i}-\bar{y}\right)}^{2}}$$

Mean Absolute Error (MAE): This measures the average magnitude of the errors in a set of predictions, without considering their direction:

15 $${MAE}=\frac{1}{n}\mathop{\sum }\limits_{i=1}^{n}\left|{y}_{i}-{\hat{y}}_{i}\right|$$

Bias: This quantifies the average prediction error:

16 $${Bias}=\frac{1}{n}\mathop{\sum }\limits_{i=1}^{n}\left({\hat{y}}_{i}-{y}_{i}\right)$$

Root Mean Square Error (RMSE): This measures the square root of the average of the squares of the errors:

17 $${RMSE}=\sqrt{\frac{1}{n}\mathop{\sum }\limits_{i=1}^{n}{\left({y}_{i}-{\hat{y}}_{i}\right)}^{2}}$$

Here, ${y}_{i}$ represents the observed sWBGT values, ${\hat{y}}_{i}$ denotes the sWBGT values calculated in this study, and $n$ is the number of observations. This method ensures a robust validation of the sWBGT data derived from the reanalysis dataset.

### Climate network validation

In our study, the climate network was validated from two perspectives: statistical significance and the reliability of network links.

First, the statistical significance of the network connections was assessed by applying the Student’s t-test to the cross-correlation between node pairs. Since cross-correlation is fundamentally a correlation coefficient, the Student’s t-test helps determine whether the correlation between two nodes is statistically significant, thereby preliminarily excluding spurious connections. After the significance tests, we found that all links passed the Student’s t-test at a significance level of 0.01, indicating a high level of statistical reliability.

Second, to ensure that the established links between nodes accurately reflect teleconnections of heat stress across regions, we conducted a reliability assessment using the following approach. We selected cities prone to extreme heat events and identified years when such events were reported. After confirming the occurrence of an extreme heat event in a specific city during a given year, we examined the connections of the corresponding node within the network. We then checked whether the regions linked to the city, particularly those located over a thousand kilometers away, also experienced extreme heat events. If two geographically distant but connected regions experienced extreme heat events within 200 days, which is the maximum lag used in the cross-correlation calculations, it was considered as evidence that the link accurately represents a teleconnection in heat stress variations.

## Data Records

The sWBGT data and data related to the climate network are accessible via figshare^66^. Except for the variables ‘lat’ and ‘lon’, all other variables are gridded data with a spatial resolution of 2° × 2°. Detailed information about each variable is recorded in Table 1. The variables currently available for download are described in Table 1. The sWBGT data are stored in the NetCDF format, which is widely used in the scientific community and can be easily read by a variety of data analysis tools. The variables related to the climate network are stored in the pickle format, which facilitates quick serialization and deserialization of Python objects.

**Table 1:** Description of variables available in the sWBGT database.

Variable Name (short)	Variable Name (long)	Description/Format	Units
sWBGT	Daily Maximum Simplified Wet-Bulb Globe Temperature	Highest simplified wet-bulb globe temperature (sWBGT) calculated from hourly data, spanning from 00 UTC to 23 UTC	°C
lat	Latitude	Geographic latitude of each grid cell	°
lon	Longitude	Geographic longitude of each grid cell	°
tau	Time Lag	Time lag corresponding to the maximum absolute value of cross-correlation	day
C_ij	Link Weights	Maximum absolute cross-correlation value between node pairs	dimensionless
W_ij	Link Strength	Link weights normalized as ${W}_{i,j}^{y}=\frac{{C}_{i,j}^{y}\left({\sigma }_{0}\right)-\left\langle {C}_{i,j}^{y}\left(\sigma \right)\right\rangle }{{std}\left({C}_{i,j}^{y}\left(\sigma \right)\right)}$	dimensionless
IN_D	In-degree	The number of edges directed towards a specific node from other nodes	dimensionless
OUT_D	Out-degree	The number of edges originating from a specific node and directed towards other nodes	dimensionless
IN_C	In-Weights	Total weights of incoming links for each node	dimensionless
OUT_C	Out-Weights	Total weights of outgoing links for each node	dimensionless
IN_W	In-Strength	Total strength of incoming links for each node	dimensionless
OUT_W	Out-Weights	Total strength of outgoing links for each node	dimensionless
DC	Degree Centrality	Combined sum of in-degree and out-degree for each node	dimensionless
AWC	Area weighted connectivity	Proportion of the node’s connection area relative to the Earth’s surface area	dimensionless
ND	Network divergence	Difference between the sum of out-degree and in-degree for each node	dimensionless

## Technical Validation

In our validation analysis, we included 1,858 stations that met the stringent criteria set forth for data completeness and variable availability. We computed the hourly sWBGT for these stations and extracted the daily maximum values to assess the accuracy of our sWBGT dataset. The validation focused on comparing these computed values against established heat stress guidelines to determine their reliability in various climatic conditions.

**Fig. 2:**
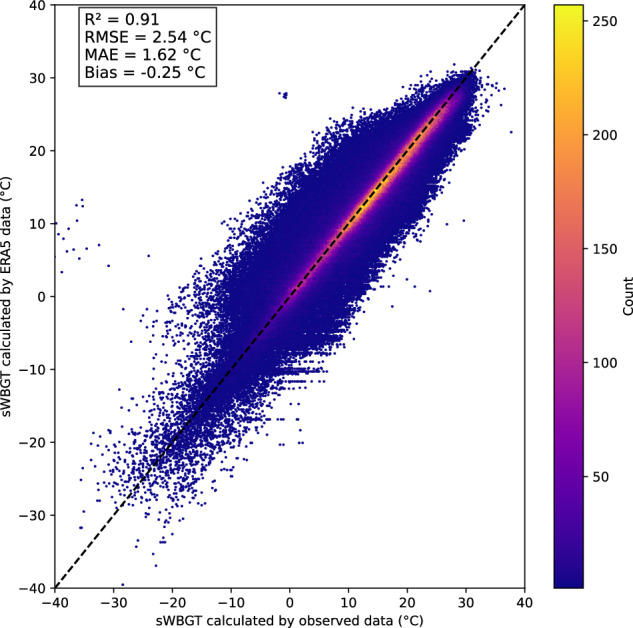
Scatter plot comparing sWBGT values calculated from observed data with those calculated from ERA5 data in 2022. Colors indicate the density of data points, and the black dashed line represents the 1:1 line.

The overall performance of the sWBGT dataset in 2022 is reflected by the R², RMSE, MAE, and mean bias values, which are 0.91, 2.54 °C, 1.62 °C, and −0.25 °C, respectively. As shown in Fig. 2, the majority of the scatter points are distributed close to the 1:1 line, suggesting a strong agreement between sWBGT values calculated using ERA5 data and those calculated using observed data. This close alignment underscores the reliability of using ERA5 data for sWBGT estimations.

**Fig. 3:**
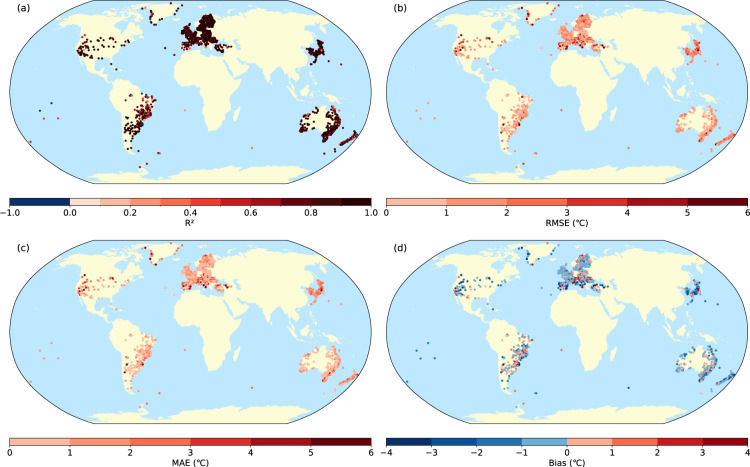
Spatial distribution of R^2^, RMSE, MAE, and bias of sWBGT at individual meteorological stations in 2022.

Figure 3 illustrates the spatial distribution of R², RMSE, MAE, and bias for sWBGT calculated from observed data versus sWBGT calculated from ERA5 across 1,858 global stations. Among these, 79.66% of the R² values exceed 0.8. Additionally, 74.11% of the RMSE values are below 2 °C, and 81.32% of the MAE values are also below 2 °C. Moreover, 83.96% of the bias values lie within ± 2 °C. The overall mean bias value is −0.25 °C, indicating a slight underestimation of the observed data by the ERA5 calculations. Spatially, there is a trend of decreasing accuracy with increasing altitude, with areas of lower accuracy predominantly located in high-altitude mountainous regions.

**Fig. 4:**
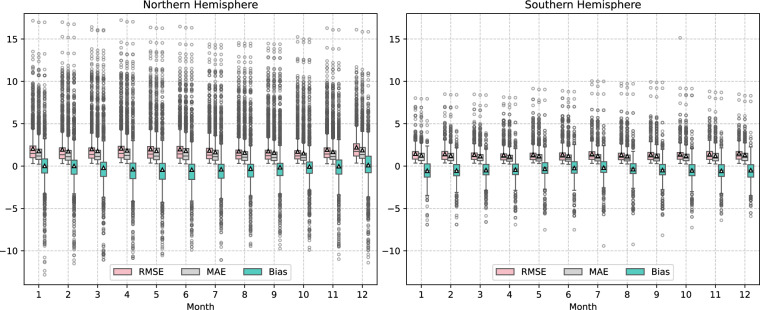
Boxplots of monthly RMSE, MAE, and Bias for sWBGT in 2022, comparing ERA5-derived values to ISD observations for the Northern Hemisphere (left) and Southern Hemisphere (right). The boxes extend from the first quartile (Q1) to the third quartile (Q3) of the data, representing the interquartile range (IQR). The black line within each box indicates the median, reflecting the central tendency of the error metrics. Whiskers extend from the boxes by a factor of 1.5 times the IQR, capturing the range of non-outlying data points. Gray circular points represent outliers, defined as values beyond Q3 + 1.5 × IQR or below Q1 - 1.5 × IQR. The mean for each metric and month is marked by a white triangle with a black outline. Colors distinguish the error metrics: red for RMSE, gray for MAE, and green for Bias, facilitating the identification of each metric’s distribution across months and hemispheres.

To assess whether the accuracy of the sWBGT dataset exhibits significant variations throughout the year, particularly in response to seasonal changes in colder and warmer months, we analyzed the monthly distribution of RMSE, MAE, and bias for sWBGT derived from ERA5 data compared to sWBGT from ISD observations in 2022. Given the reversed seasonal patterns between the Northern and Southern Hemispheres, calculating global monthly averages could mask hemispheric differences in seasonal error variations. Therefore, we computed these error metrics for each hemisphere and month, as shown in Fig. 4. The boxplots reveal that, for both the Northern and Southern Hemispheres, the three error metrics (RMSE, MAE, and bias) exhibit relatively stable seasonal variations without dramatic fluctuations. Overall, the Southern Hemisphere shows slightly smaller box sizes, indicating a narrower range of error variability compared to the Northern Hemisphere. The distributions of RMSE and MAE remain closely aligned, suggesting consistent and uniform error distribution across months. The bias boxplots tend to skew toward negative values, indicating a potential systematic underestimation of sWBGT from ERA5 data relative to sWBGT from ISD observations.

**Fig. 5:**
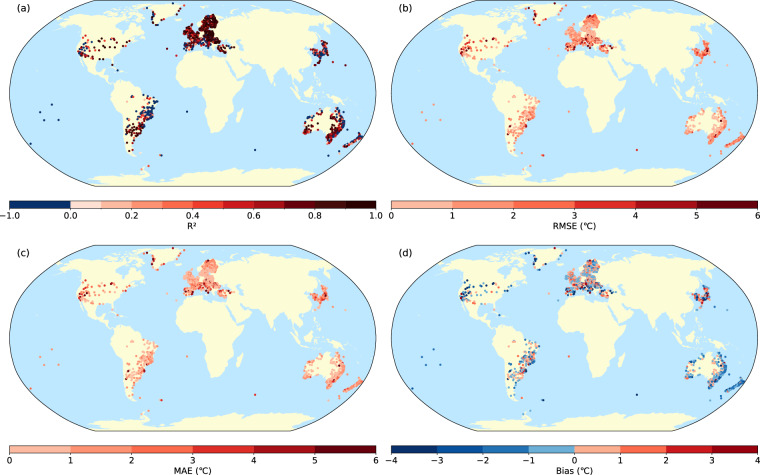
Spatial distribution of R², RMSE, MAE, and bias of sWBGT at individual meteorological stations in January 2022, based on daily data from that month.

Building on the monthly distribution analysis in Fig. 4, we introduce Fig. 5 to provide a site-specific evaluation of the calculated sWBGT for January 2022, presenting the spatial distribution of four statistical metrics: R², RMSE, MAE, and bias at individual meteorological stations based on daily data from that month. This figure complements Fig. 4 by offering a detailed site-level comparison of sWBGT derived from ERA5 data against measurements from ISD observations, highlighting the spatial variability in sWBGT dataset performance for a specific month. The distribution indicates that, despite the limited time series, the calculated sWBGT maintains reliable performance, with R² values generally showing moderate to strong correlations, while RMSE and MAE values align with the monthly trends observed in Fig. 4, and bias values suggest a slight underestimation, consistent with the negative skew noted earlier. This site-specific analysis for January 2022 confirms the robustness of our sWBGT calculations across diverse locations, supporting their applicability for short-term, site-specific assessments.

We evaluated the reliability of our climate network by focusing on two documented extreme heat stress events in Karachi, Pakistan (June 2015), and Changsha, China (summer 2020). These cities experience pronounced heat stress due to their subtropical climates and significant population densities^67,68^.

**Fig. 6:**
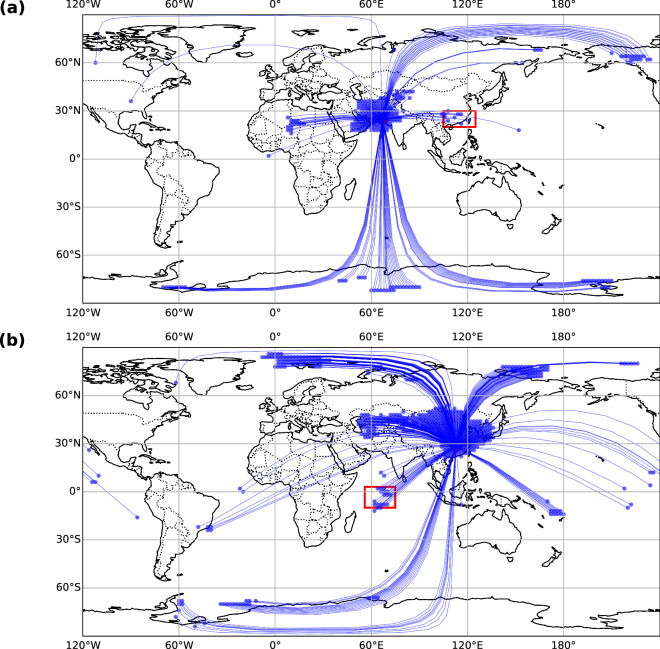
Connectivity of nodes in the climate network. (**a**) Node connections for Karachi in 2015 showing its interactions within the network. (**b**) Node connections for Changsha in 2020 illustrating its linkages within the network. Red circles represent Karachi and Changsha, blue circles denote connected nodes, and blue arcs indicate the links between nodes. Red squares highlight the regions of Southern China and the tropical Indian Ocean.

In June 2015, an intense heatwave in Karachi claimed over 1,180 lives, primarily among residents with limited access to cooling and outdoor workers^69,70^. In our 2015 climate network (Fig. 6a), the Karachi node was not only connected to nearby regions but also formed multiple links with distant areas such as southern China, the Arctic, and Antarctica. We further investigated temperature variations in these regions during 2015. According to China’s climate bulletin, southern China experienced frequent extreme heat events in the summer of 2015^71^. The Arctic underwent exceptionally high temperature anomalies in 2015, with December 2015 to February 2016 marking the warmest winter on record there^72,73^. Meanwhile, on March 24, 2015, a record-high temperature of 17.5 °C was observed at Esperanza Station on the northern tip of the Antarctic Peninsula^74^. These phenomena suggest that our network links indeed captured real-world co-occurrences or sequential occurrences of heat stress across these remote areas.

During the summer of 2020, Changsha experienced alternating periods of heavy rainfall and extreme heat, resulting in persistently hot and humid conditions^75^. In the 2020 network (Fig. 6b), the Changsha node was connected not only to neighboring areas in eastern and southern China but also to more distant regions such as Mongolia, Central Asia, and the equatorial Indian Ocean. According to the Mongolian National Agency for Meteorology and Environmental Monitoring, 2020 was among the hottest years on record in Mongolia since 1940^76^. Kazakhstan faced record-breaking temperatures, and Tashkent, Uzbekistan, suffered a severe heatwave^77,78^. Research also indicates that unusually high sea surface temperatures persisted in the tropical Indian Ocean during early summer 2020^79^. This further demonstrates that our network can detect spatiotemporal correlations of heat stress across geographically distant regions.

By examining these well-documented heatwaves in Karachi and Changsha and verifying those far-flung connected nodes that indeed experienced elevated heat, we provide evidence that the climate network can effectively detect and map out significant spatiotemporal correlations in global heat stress. Nonetheless, while the network identifies these correlations, a confirmed physical mechanism requires further analysis, especially for the most distant links.

A notable example of an established physical linkage within our network is that between southern China and the tropical Indo-Pacific region. Previous study has demonstrated that warming in the tropical Indian Ocean, along with rapid transitions from El Niño to La Niña phases in the central-eastern tropical Pacific, can induce large-scale circulation anomalies, specifically, a westward-extending Western North Pacific Subtropical High and enhanced anticyclonic circulation over southern China^79^. These circulation patterns reduce cloud cover, intensify subsidence, and thereby sustain extreme heat stress in southern China, illustrating how the climate network’s links in this region are not merely statistical but supported by observational and model-based research.

In summary, our climate network approach, which uses statistical measures across gridded sWBGT data, successfully identifies pairs of regions exhibiting closely related heat stress variations. Validation with real-world events in Karachi (2015) and Changsha (2020) indicates that many distant but connected nodes did co-experience extreme temperatures within the defined correlation lag window, reflecting global-scale patterns of heat stress concurrency. However, while certain links, such as that between southern China and the tropical Indo-Pacific, have a well-established physical mechanism, other connections (for example, those involving Karachi and polar or mid-latitude regions) currently lack a clear mechanistic explanation. Further targeted research, including advanced modeling studies, will be necessary to clarify the dynamic pathways that might underlie these less-understood teleconnections.

By distinguishing links supported by known processes from those that remain statistically significant yet mechanistically uncertain, we encourage further investigations aimed at refining our understanding of how heat stress extremes propagate or cluster worldwide. Existing studies have demonstrated that, under various future global climate scenarios, land systems in certain terrestrial tipping elements and densely populated regions will undergo significant changes^80–82^. These shifts present new challenges for analyzing heat stress teleconnections and addressing them will be crucial for informing risk management, preparedness, and adaptation strategies at both local and international scales.

## Data Availability

Python scripts provided by Li *et al*.^32^ to calculate hourly sWBGT from ERA5 data are available on GitHub (https://github.com/dw-li/WBGT). In constructing our climate network, we referenced the code by Liu *et al*.^53^ (https://github.com/fanjingfang/Tipping). We have adjusted this code to adapt it to our global-scale research and the calculation of various network metrics. The relevant scripts have also been uploaded to figshare^66^.
